# Assessing the Association Between Internet Addiction Disorder and Health Risk Behaviors Among Adolescents and Young Adults: A Systematic Review and Meta-Analysis

**DOI:** 10.3389/fpubh.2022.809232

**Published:** 2022-04-01

**Authors:** Jun Wang, Qing-hong Hao, Yang Tu, Wei Peng, Yang Wang, Hui Li, Tian-min Zhu

**Affiliations:** ^1^School of Rehabilitation and Health Preservation, Chengdu University of Traditional Chinese Medicine, Chengdu, China; ^2^School of Acupuncture and Tuina, Chengdu University of Traditional Chinese Medicine, Chengdu, China; ^3^School of Preclinical Medicine, Chengdu University, Chengdu, China

**Keywords:** internet addiction disorder, systematic review, association, adolescents and young adults, health risk behaviors

## Abstract

**Background:**

Internet addiction disorder (IAD) is a global issue that has resulted in a slew of physical and emotional consequences. Studies have indicated that health risk behaviors might be the risk factors for IAD. The published literature on the correlation between the two is lacking. Therefore, we conducted a comprehensive analysis to understand better the link between IAD and health risk behaviors among adolescents and young adults.

**Methods:**

We searched ten electronic databases for relevant articles. Data were extracted based on IAD and health risk behaviors ( alcohol, smoking, suicidal, gambling and drug abuse). We calculated odds ratios (ORs), a pooled correlation coefficient (r) and 95% confidence intervals (CIs). A fixed-effect model was applied to summarize the pooled effects. Heterogeneity was examined using *I*^2^ statistics and Cochran's Q statistics. All analyses were conducted by using Stata version 15.0.

**Results:**

A total of 16 studies and 61,823 participants were included in this study. Meta-analysis showed that IAD was positively correlated with drinking (*r* = 0.35; 95% CI 0.32–0.37) and smoking (*r* = 0.12; 95%CI 0.10–0.15), and was associated with an increased risk of suicidal behavior (OR= 1.95; 95% CI 1.65–2.30), drinking (OR= 1.75; 95% CI 1.65–1.85), and smoking (OR= 1.63; 95% CI 1.54–1.72) among adolescents.

**Conclusion:**

We found significantly increased risks of suicidal behavior, drinking, and smoking in adolescents and young adults with IAD. These findings are important to expand our understanding of IAD and have great guiding significance for preventing health risk behaviors of adolescents and young adults.

**Systematic Review Registration:**

https://www.crd.york.ac.uk/prospero/display_record.php?ID=CRD42021257729, identifier: PROSPERO CRD42021257729.

## Introduction

Internet addiction disorder (IAD) is considered as an inability of individuals to control their internet use, resulting in marked distress and functional impairment in daily life such as psychological, social, academic, and professional problems (1). The overall prevalence of IAD ranges from 1.5 to 8.2% in the United States and Europe (2). IAD has attached much attention throughout the world among adolescents, with a prevalence of 7.9–16.0% (3–5).

To date, the diagnostic criteria, and classification of IAD are still controversial, and its assessment instruments are inconsistent due to sociocultural and socioeconomic factors. Some scholars have argued that using traditional addiction criteria to define the boundaries of the IAD does not highlight the uniqueness of the behavior itself (6, 7). It has been suggested that IAD is not a mental disorder or addiction, but rather a problematic behavior (8, 9). To date, its causative theories and clinical features are still under investigation and being constantly updated.

Notably, internet gaming disorder (IGD) is an important subtype of IAD (10). IGD as a proposed behavioral addiction included in Section III of DSM-5 (11) and adopted at the World Health Assembly as a diagnosis in ICD-11 (12). The number of adolescents with IGD who suffer withdrawal and tolerance symptoms and lose interest in other activities is increasing dramatically and has now been recognized as a common behavioral problem worldwide (13).

Risky behavioral lifestyles, such drinking, smoking, suicidal behavior, gambling and drug abuse are closely related to the healthy development of adolescents and young adults. Previous research reported a positive association between IAD and adolescents Risky behavioral lifestyles, such drinking, smoking, suicidal behavior, gambling and drug abuse are closely related to the healthy development of adolescents and young adult's risk behaviors (14, 15). Gansner et al. (16) and Berardelli et al. (17) found that the level of internet dependence was associated with a higher suicidal behavior risk. Kim et al. (18) and Na et al. (19) showed that the time spent playing internet games is closely related to the degree of alcohol consumption, but Poorolajal et al. (20) found no significant correlation between the two. Some studies have suggested that smokers might more easily develop IAD (21, 22). Furthermore, a study on 467 Chinese adolescents demonstrated that individuals with IAD or IGD might engage in more risky behaviors (skipping school, smoking, drinking, fighting, gambling, stealing) (23).

Taken together, the data from this series of studies have been useful in demonstrating that IAD is associated with health risk behaviors. However, due to the limitations of the sample sizes and regional effects in many studies, their results were different to some extent. Thus, it is necessary to evaluate the relationship between IAD and health risk behaviors. The findings will lay the foundation for studying the characteristics of IAD among adolescents and young adults and its associated correlates. The results may provide evidence to support the development of more effective interventions.

## Materials and Methods

This systematic review and meta-analysis were conducted following the Preferred Reporting Items for Systematic Review and Meta-Analyses (PRISMA) (24, 25) guidelines. The review protocol was registered in PROSPERO (CRD42021257729) URL: https://www.crd.york.ac.uk/prospero/#recordDetails.

### Search Strategies

We searched ten international electronic databases including PubMed, Embase, Web of Science (WOS), the Cochrane Library, PsycINFO, ERIC, China National Knowledge Infrastructure (CNKI), Chinese Biomedical Literature Database (CBM), Technology Periodical Database (VIP) and Wan Fang Database. We also performed a manual search of the gray literature listed in the bibliography, including dissertations and conference papers. This search was updated on Aug 2021. The detailed search strategy for PubMed is provided in Supplementary Material 1, and other databases are modified as necessary.

### Eligibility Criteria

#### Study Design

Studies were included if they fulfilled the following criteria: (1) used a validated scale to assess IAD; (2) reported the relationship between IAD and health risk behaviors; (3) were observational studies published up to Aug 2021; (4) had a Pearson or Spearman correlation coefficient (r) or odds ratios (ORs) available; (5) were published in Chinese or English.

Case reports, meeting abstracts, review papers, commentaries, and those with inadequate information were excluded.

#### Participants

Participants were adolescents and young adults aged between 12 and 25 with a standard diagnosis of IAD and associated health risk behaviors. There were no restrictions on gender or race.

#### Exposures

Studies needed to have clearly reported smoking, drinking, suicidal behavior and other health risk behaviors. For the purposes of this review, health risk behaviors were defined as related behaviors reported at least once in the previous 30 days. That is, a study presenting results in terms of a range of frequencies (“often” and “frequently”) would be chosen for inclusion.

#### Interventions and Comparisons

Since this meta-analysis was based on published articles to investigate the relationship between IAD and health risk behaviors, there were no comparison or intervention groups.

#### Outcome Measures

Studies must have included IAD as an independent variable and health risk behaviors as dependent variables for identified associations. Studies that did not adjust for these variables in the outcome analysis were included in this study. Health risk behaviors were measured by the related scales or self-report questionnaires. IAD was measured by Young Diagnostic Questionnaire for IAD (YDQ) (26), Chinese Internet Addiction Scale Revision (CIAS-R) (27), Internet Addiction Test (IAT) (28) and other relatively high-quality scales/questionnaires designed to measure IAD.

### Data Extraction

Two reviewers independently (JW and QH) completed the title, abstract and full text screening. Items were extracted from the eligible studies including: first author, publication year, country, sample size, age, gender, measures of IAD and health risk behaviors, Pearson or Spearman correlation coefficient (r) or ORs between IAD and health risk behaviors. Disagreements were settled by consensus and discussion with a third reviewer (YT).

### Quality Assessment

The Agency for Healthcare Research and Quality (AHRQ), which contains 11 items, was used to evaluate the quality of the cross-sectional studies. The answer “yes” scored 1, while “no” or “unclear” scored 0. Scores of 8–11 were classified as high-quality research, and scores of 4–7 were classified as moderate quality (29).

### Data Synthesis and Analysis

The relationship between IAD and health risk behaviors was assessed using the Pearson correlation coefficient (r- value). “Fisher's z transformation” was used to convert Spearman's correlation coefficients into the normal distribution. The formula for the transformation is z = 0.5[ln(1+*r*)−ln(1−*r*)] where “ln” is the natural logarithm (30). In addition, the included studies were weighted according to the magnitude of the respective standard error (SE). The formula for the transformation is SE = $\sqrt{N-3}$ where “N” represents the respective sample size (30). The degree of variation was estimated by the standard error (SE) and 95% confidence intervals (CIs). The *r* < 0.21 indicated poor correlation; 0.21 ≤ *r* < 0.41 was considered average correlation; 0.41 ≤ *r* < 0.61 suggested moderate correlation; 0.61 ≤ *r* < 0.81 meant significant correlation and > 0.81 suggested strong correlation (31). For meta-analyses of ORs, we used the logarithm as the effect size (32). Final results were transformed from the log of the ORs to the ORs for presentation. Heterogeneity was determined using *I*^2^ statistics and Cochran's Q statistics. A fixed-effects model was employed to analyze the data if *I*^2^ ≤ 50% and the *P*-value for the Q-statistic > 0.05. Otherwise, a random-effects model was applied (33). If notable heterogeneity was found, a sensitivity analysis was performed and we examined the stability of the pooled estimate for each study by excluding individual studies one by one from the analysis. Publication bias was examined by Egger's tests and funnel plots (34). The statistical significance was set at *P* < 0.05.

All of the above data processing was conducted with STATA Version 15.0 Statistical Software.

## Results

### Selected Studies

**Figure 1:**
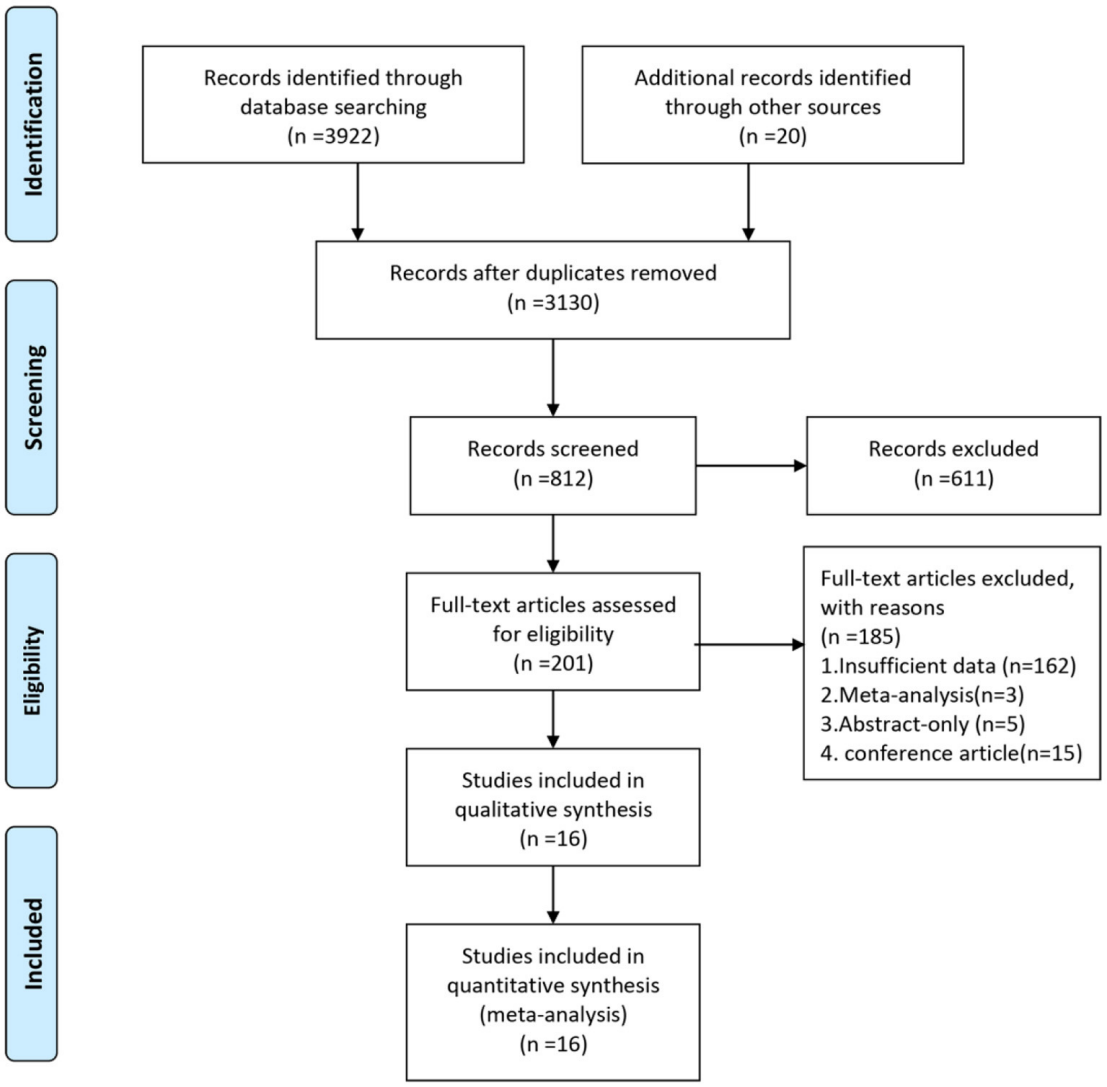
PRISMA flowchart of included articles.

A total of 3,942 records were identified, and 3,130 records were left after removing duplicates. Finally, 201 records were needed for full-text assessments after reviewing the titles and abstracts. After reading the full-text articles, 16 studies were selected for inclusion in this systematic review. The study selection process is displayed in Figure 1.

### Study Design Characteristics

Table 1 shows the characteristics of the 16 included studies. All included studies were cross-sectional studies. A total of 61,823 participants were included in this review. The included studies were conducted in more than seven different countries around the world, including the US (*n* = 1), Switzerland (*n* = 1), Iran and several other middle eastern countries (*n* = 1), Ethiopia (*n* = 1), Lebanon (*n* = 2), Spain (*n* = 2), and China (*n* = 8). Although several studies did not specify the age range of the participants, they mentioned the educational stage of the participants, and the majority of them were university students. IAT is the most commonly used diagnostic tool for IAD, followed by YDQ. Regarding the measures of health risk behaviors, most of them were questionnaires/scales drawn up based on the cultural background of the country.

**Table 1:** Characteristics of the included studies.

**Reference**	**Sample size**	**Age range**	**Nation**	**Assessment (IAD)**	**Assessment (Problem behaviors)**	**Study quality**
Pallanti et al. (35)	200	15–16	US	IAS	SPQ	7
Zhang (36)	1,086	N/A	China	YDQ	YRBSQ	7
Li (37)	3,637	N/A	China	YDQ	Self-defined questionnaire	8
Tang (38)	1,275	(19.81 ± 1.006) 18–25	China	YDQ	Adolescent health risk behavior surveillance questionnaire	7
Dong (39)	3,719	N/A	China	YDQ	Investigation plan on health-related behaviors of Chinese adolescents	8
Rücker et al. (40)	3,077	14.2	Switzerland	The French version of the IAT	Questionnaires about substance use	7
Zhang (41)	1,561	(19.16 ± 1.821)	China	IADS	Investigation report on health risk behaviors of Chinese urban adolescents	6
Yan et al. (42)	1,282	(19.1 ± 1.1)	China	IAT	FTND AUDIT	5
Zhang et al. (43)	1,091	N/A	China	Questionnaire on Health Related/Dangerous Behaviors of Chinese Adolescents	Questionnaire on health related/dangerous behaviors of Chinese adolescents	6
Zhu (44)	268	N/A	China	Mobile Phone Dependence Scale for Middle School Students	Adolescent health-related behavior survey questionnaire	7
Poorolajal et al. (20)	4,261	N/A	Iran	The PIU-15 questionnaire	The GHQ-28 questionnaire	6
Fernández-Aliseda et al. (45)	35,370	14–18	Spain	CIUS	Questionnaire about variable substance consumption	6
Laurette et al. (46)	1,810	14–17	Lebanon	IAT	AUDIT FTND	8
Zenebe et al. (47)	603	N/A	Ethiopia	IAT	The K10-item scale	6
Ramón-Arbués et al. (48)	698	N/A	Spain	IAT	the CAGE questionnaire	6
Dib et al. (49)	1,810	14–17	Lebanon	IAT	AUDIT	7

*AUDIT, Alcohol Use Disorders Identification Test; CIAS, Chen Internet Addiction Scale; CIAS-R, Revised Chen Internet Addiction Scale; CIUS, the Compulsive Internet Use Scale; FTND, Fagerström Test for Nicotine Dependence; IADS, Internet Addiction Diagnostic Scale; IAS, the Internet Addiction Scale; IAT, The Young Internet Addiction Test; SPQ, The Shorter PROMIS Questionnaire; YDQ, the Young's Diagnostic Questionnaire; YRBSQ, Youth Risk Behavior Survey Questionnaire*.

### Quality of the Studies

All included studies scored above four points, ranging from 5 to 7 (Table 1).

### Meta-Analysis

#### The Association Between IAD and Health Risk Behaviors According to Pearson Correlation

**Figure 2:**
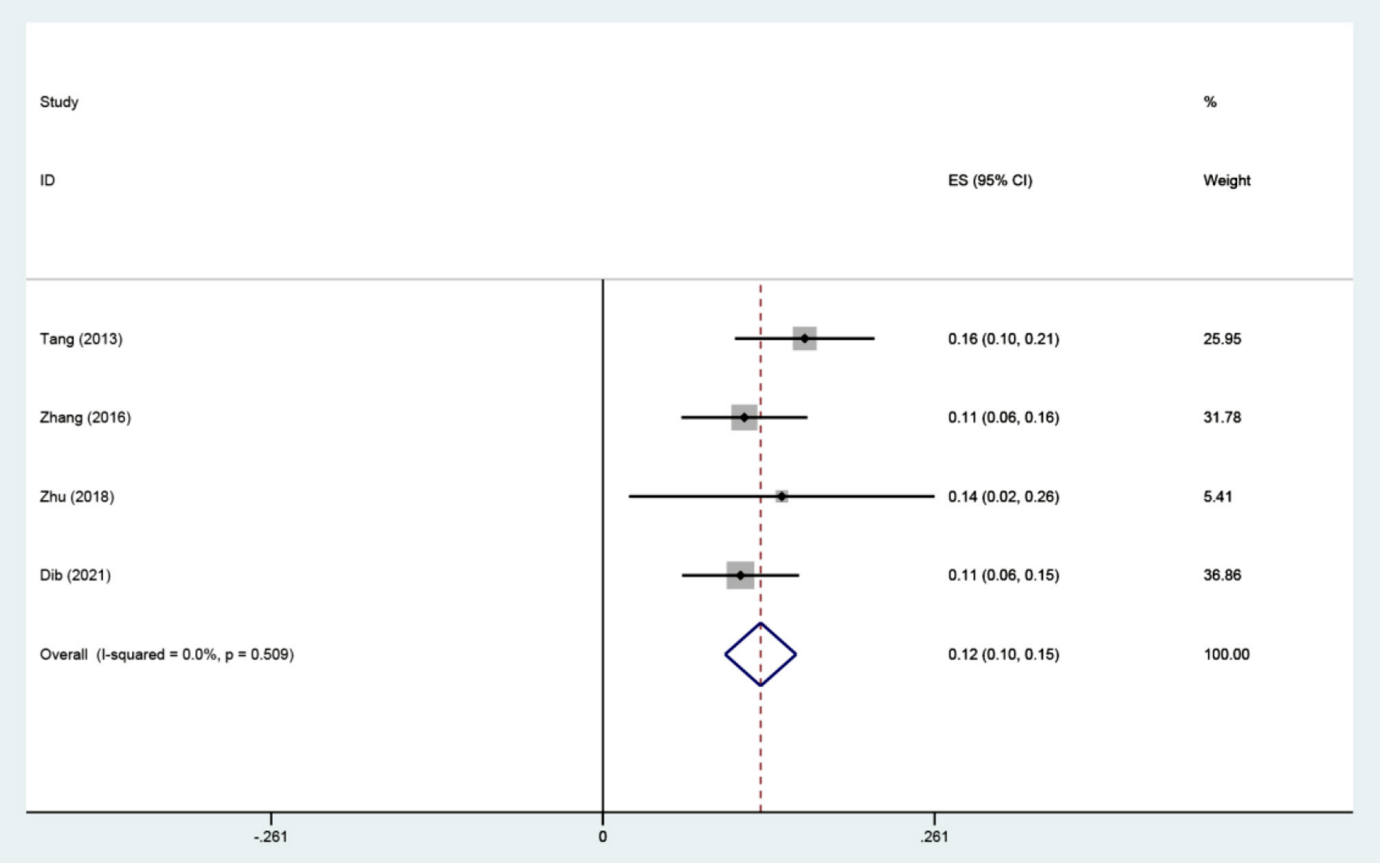
The association between IAD and drinking (r).

**Figure 3:**
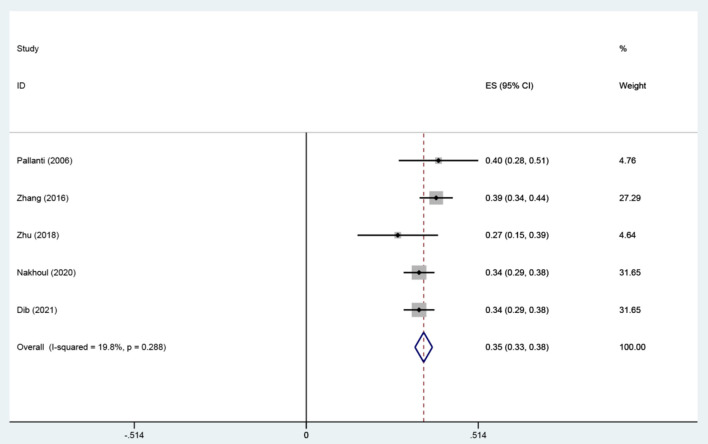
The association between IAD and smoking (r).

**Figure 4:**
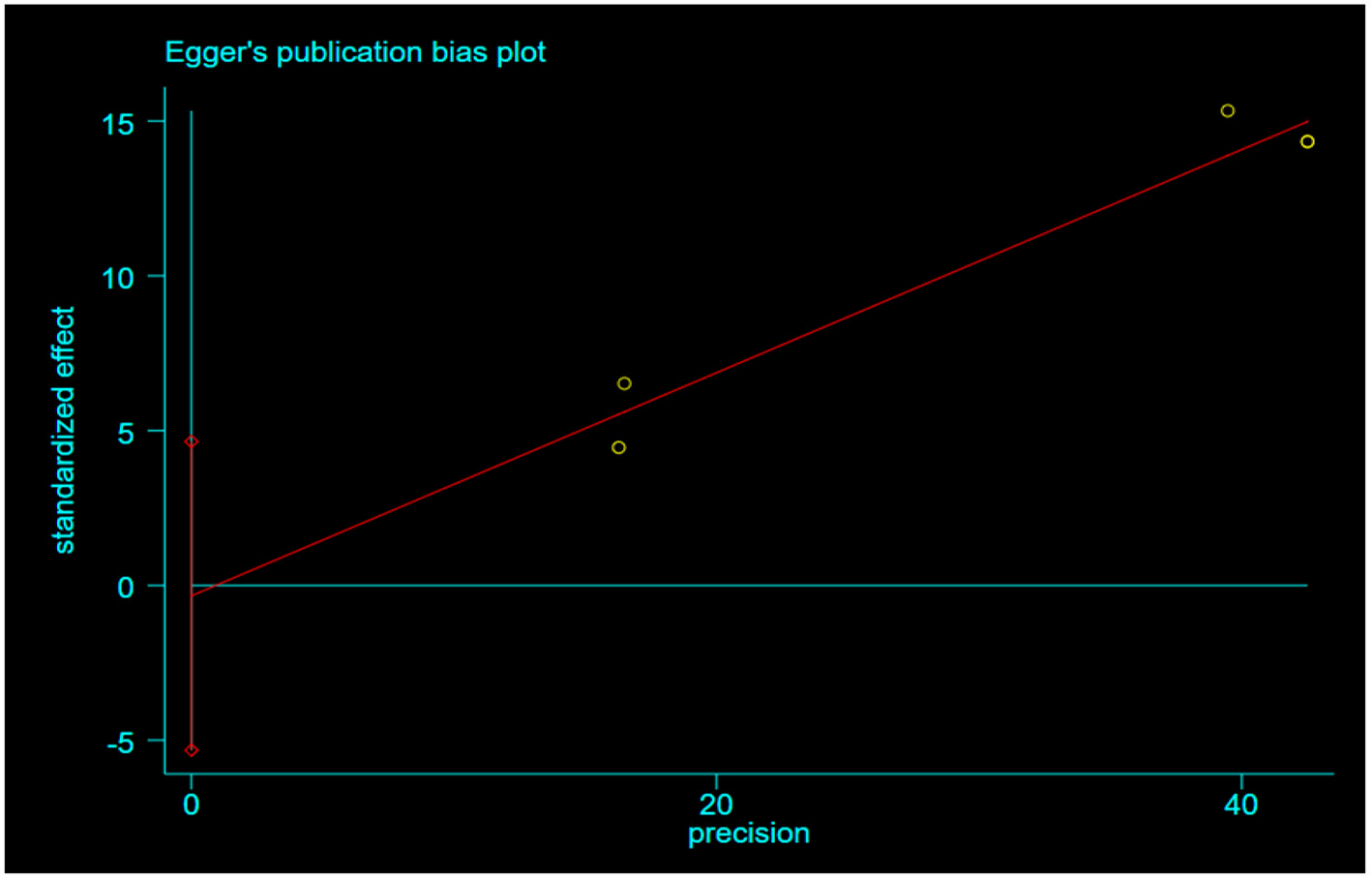
Egger's publication bias plot for the association between IAD and smoking.

Seven studies were included to assess the relationship between IAD and smoking (36, 38, 41, 44, 49), drinking (35, 41, 44, 46, 49). There were positive associations between IAD and drinking, and smoking. A fixed-effects model was used to calculate the effect size of IAD and drinking (*I*^2^= 19.8%, *P* = 0.288). The combined correlation coefficient(r) was (*r* = 0.35; 95% CI 0.32, 0.37) (Figure 2). The combined effect size of IAD and smoking was (*r* = 0.14; 95% CI 0.10, 0.18) with slight heterogeneity (*I*^2^= 55.6%, *P*= 0.061). We conducted sensitivity analyses to identify the source of the heterogeneity. The result indicated that heterogeneity was significantly reduced when we removed the study by Zhang (36). We found that Zhang's study investigated the students whose former-drinkers accounted for the largest proportion, while the others focused on current drinking. We recalculated the effect size based on the fixed-effects model after excluding this study (*r* = 0.12; 95% CI 0.10, 0.15) and the heterogeneity disappeared (*I*^2^ = 0.00%, *P* = 0.509) (Figure 3). No apparent publication bias was observed according to Egger's test (*P* = 0.844) (Figure 4).

#### The Association Between IAD and Health Risk Behaviors According to Logistic Regression

**Figure 5:**
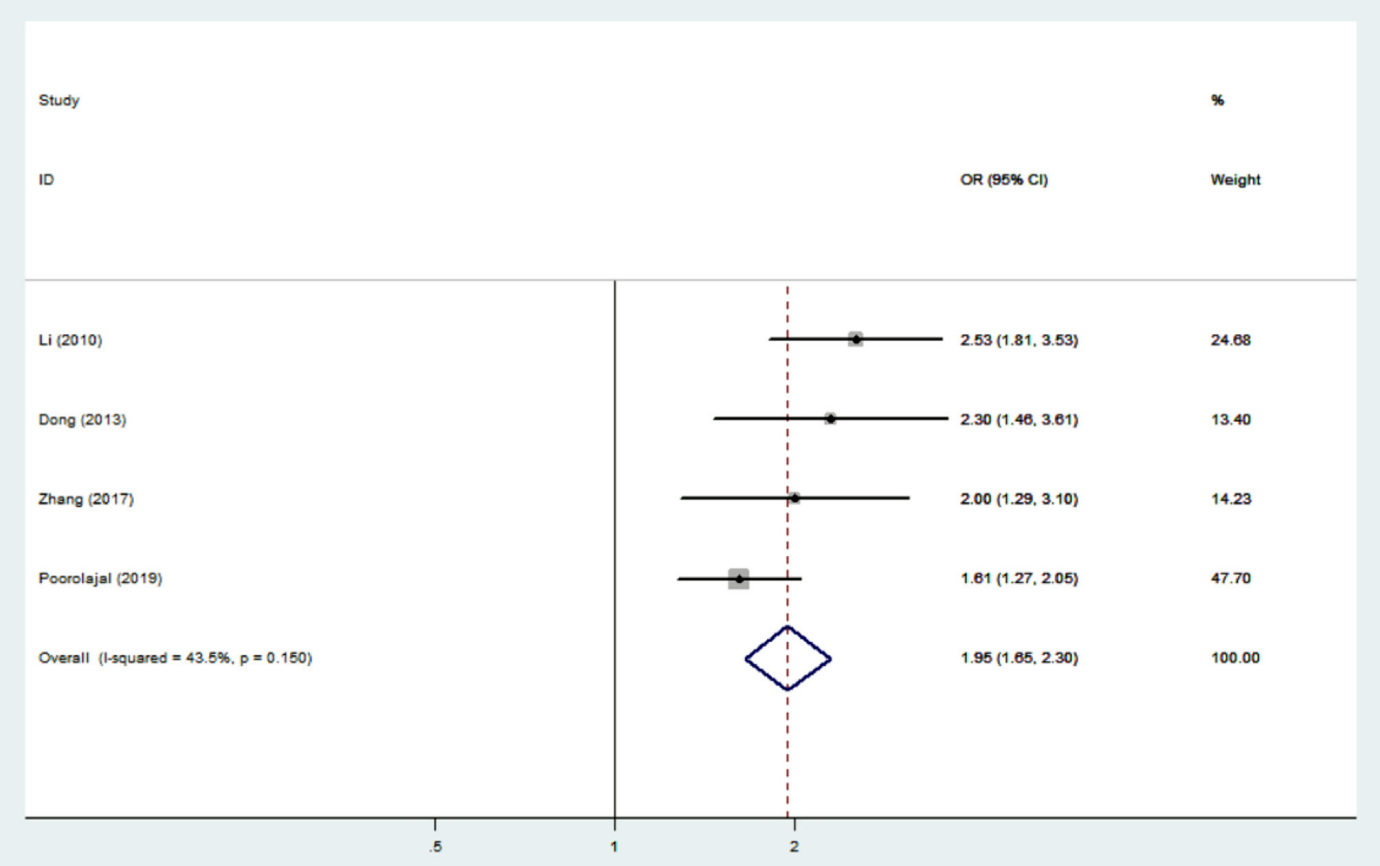
Effect of IAD on suicidal behavior (OR).

**Figure 6:**
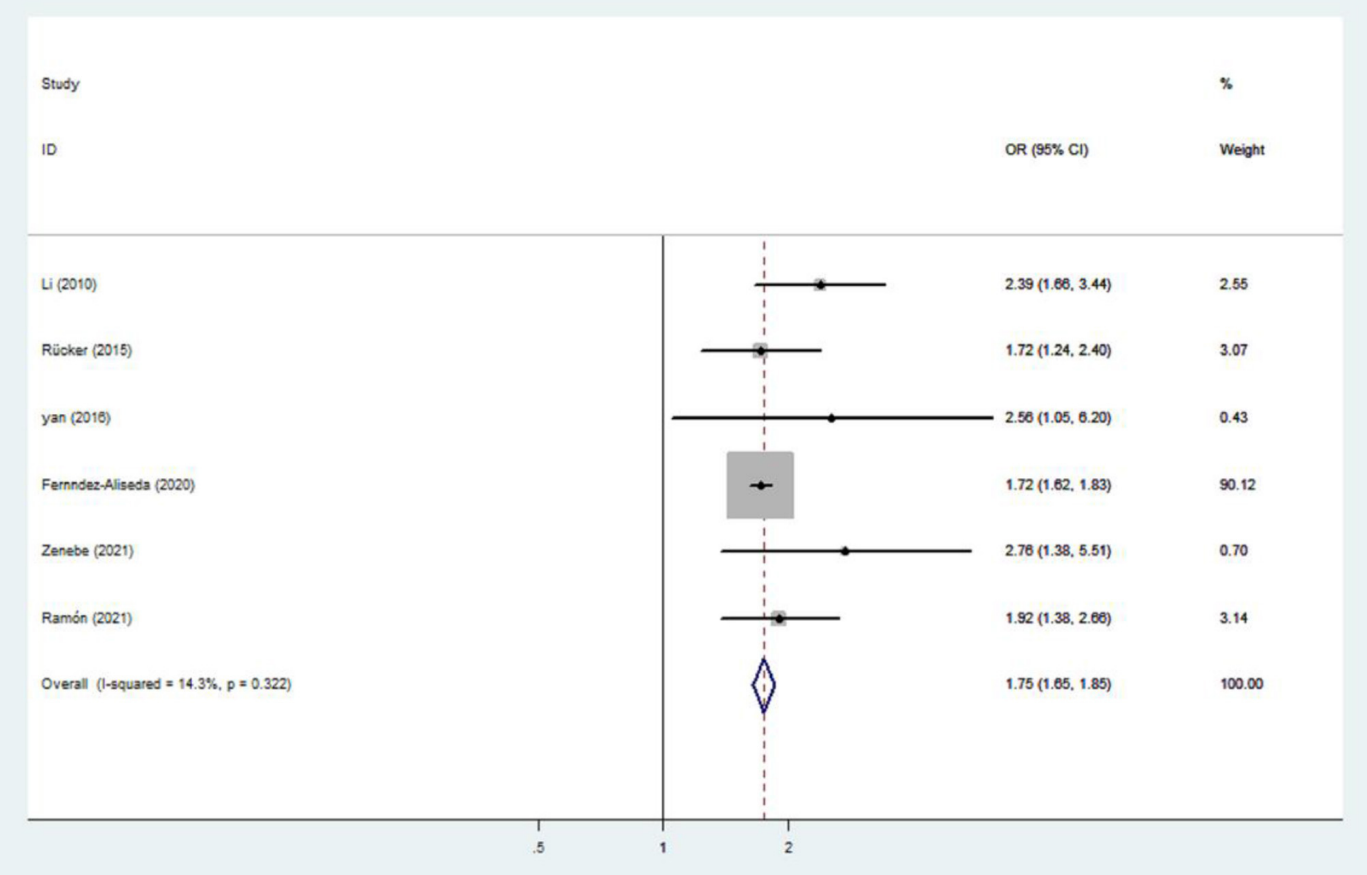
Effect of IAD on drinking (OR).

**Figure 7:**
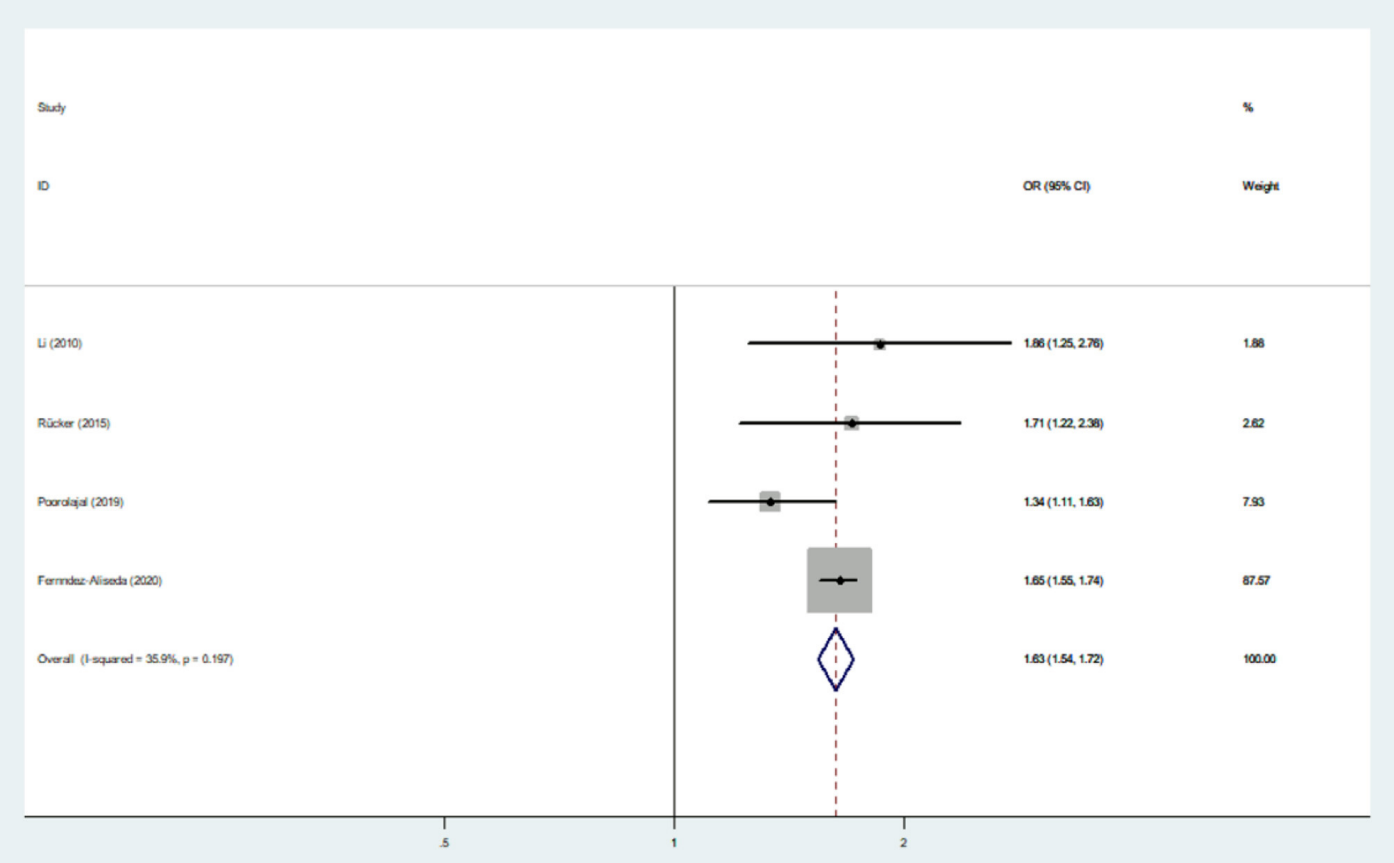
Effect of IAD on smoking (OR).

Nine studies were used to evaluate the association between IAD and smoking (20, 37, 40, 45), suicidal behavior (20, 37, 39, 43) and drinking (37, 40, 42, 45, 47, 48). The heterogeneity was not significant (*I*^2^ < 50%), and a fixed-effects model was applied. The sizes of the pooled effect of IAD on suicidal behavior, drinking and smoking were (OR = 1.95; 95% CI 1.65–2.30) (Figure 5), (OR= 1.75; 95% CI 1.65–1.85) (Figure 6) and (OR = 1.63; 95% CI 1.54–1.72) (Figure 7), respectively.

## Discussion

Our meta-analyses showed that IAD was positively associated with smoking and drinking. We found that IAD was significantly associated with an increased risk of suicidal behavior, drinking and smoking.

The present review found that adolescents and young adults with IAD have a significantly higher risk of suicidal behavior than others, in line with the literature (50–52). A total of 4 studies with 12,708 participants were included. All of the participants were students, and one study only investigated all ethnic minority students living in rural areas, which limited the generalizability of the findings (43). In addition, all included studies highlighted mood disorders, the broadest contributing factor to suicidal ideation in adolescents, consistent with previous studies (53). Adolescents and young adults have more negative self-cognition and are prone to mood disorders when facing pressure (20, 37, 39, 54, 55), and rural students who lack family care, supervision, and opportunities to share their distress, are more likely to have negative emotions and suicidal ideation (43).

The internet is a virtual space with identity concealing that can provide an online sharing community, coupled with the poor self-control of adolescents and young adults, which makes them more likely to develop IAD (20, 37, 39, 56). At the same time, long-term exposure to such negative information increases the risk of suicidal behavior (57). In conclusion, it is of great significance to pay attention to the emotional state of adolescents and carry out necessary interventions for the prevention of IAD and suicidal behavior.

There were 13 studies with 55,927 participants included that evaluated the relationship between IAD and drinking and smoking. The results suggested that there was a average positive correlation between IAD and drinking, and a poor positive correlation with smoking. Analysis based on ORs showed that IAD was significantly associated with an increased risk of drinking and smoking.

Considering the differences in sociocultural and economic background among populations, there is no standard recognized assessment tool for health risk behavior assessment at present, and many studies are based on self-report questionnaires prepared by researchers . Some studies included data on engaging in at least once health risk behaviors in the past 30 days (20, 38, 40, 41, 45, 48), some included data on the use of relevant questionnaires to assess health risk behaviors (35, 42, 44, 46, 47, 49), and others only included data on often or frequent occurrences of health-risk behaviors (37), which reduced the accuracy of the results. The assessment tools for health risk behaviors should be more standardized in the future.

Additionally, scholars have found that smoking and drinking usually cluster since they may serve the same purpose socially and psychologically (58, 59). Researchers also noted that adolescent and young adults use of multiple different substances is associated with sensation seeking, risk-taking and increased experimentation (58, 60, 61), which are also among the motivations for playing online games (62, 63). Moreover, as previous research has demonstrated, internet activity, gaming cues, nicotine and alcohol all alter the brain's neural networks, activating brain regions involved in reward and motivational processing, such as the striatum, insula, and anterior cingulate cortex, resulting in abnormal metabolism of dopamine, which affects the function of the reward system (22, 64–66). This may also explain to a certain extent the increased risk of smoking and alcohol consumption in participants with IAD or IGD.

IAD has become an important global mental health issue, and is closely associated with an increased risk of drinking and smoking. Suicidal behavior is a risky behavior that interacts with and influences IAD. Adolescence, is at a stage when individuals are easily influenced by various complex and tempting social environments, forming a series of health risk behaviors (67). Therefore, prevention and education programs are urgently needed. Schools, teachers, and parents should pay more attention to the mental and emotional health of students and provide more timely practice guidance. In addition, governments should provide safety-net mental health services to adolescents and young adults when needed.

The limitations of this review were as follows: first, there were significant differences among reports on health risk behaviors due to the huge sociocultural context among different racial/ethnic groups that had implications for the current review. Second, suicidal behaviors includes suicidal ideation, suicide planning and suicide attempts. Since suicidal ideation is a strong risk factor for suicidal behaviors (68), we extracted data related to suicidal ideation. Finally, previous research showed that drinking and smoking were higher among boys, while girls had higher rates of suicidal behavior (69–71). Unfortunately, the data extracted in this study did not allow any gender associations to be analyzed. In addition, we also searched for articles about the relationship between IAD and drug misuse and gambling, but most existing literature only concerns online gambling and specific drug abuse.

## Conclusion

Adolescents and young adults with IAD are at higher risk for suicidal behavior, drinking and smoking. Mood disorders are important mediators between IAD and suicidal behavior, and may be a target for effective intervention. In addition, it is important to explore the neural mechanisms of IAD/IGD and smoking, drinking and other substance dependence for the prevention and intervention of those problematic behaviors. The findings of this review are important for educational and psychological practitioners, parents and schools, and may provide guidance for the prevention and intervention of IAD and health risk behaviors.

## Data Availability Statement

The original contributions presented in the study are included in the article/Supplementary Material, further inquiries can be directed to the corresponding authors.

## Author Contributions

JW and QH conceived the idea. JW drafted the manuscript. YT, WP, and HL were involved in the interpretation of the study findings. TZ and YW reviewed the manuscript and provided comments. All authors contributed to the article and approved the submitted version.

## Funding

This research was funded by the Natural Science Foundation of China (81072852 and 81574047), the Key R&D Project of Sichuan Province (2019YFS0175), the Xinglin Scholar Research Promotion Project of Chengdu University of TCM (XSGG2019007), and the Training Funds of Academic and Technical Leader in Sichuan Province.

## Conflict of Interest

The authors declare that the research was conducted in the absence of any commercial or financial relationships that could be construed as a potential conflict of interest.

## Publisher's Note

All claims expressed in this article are solely those of the authors and do not necessarily represent those of their affiliated organizations, or those of the publisher, the editors and the reviewers. Any product that may be evaluated in this article, or claim that may be made by its manufacturer, is not guaranteed or endorsed by the publisher.
