# Supplementary material for: Assessing the Association Between Internet Addiction Disorder and Health Risk Behaviors Among Adolescents and Young Adults: A Systematic Review and Meta-Analysis
Source: Front Public Health. 2022 Apr 1;10:809232. doi: 10.3389/fpubh.2022.809232 (PMC9010676; doi:10.3389/fpubh.2022.809232)
Supplement: Supplementary file 1 [file Table_1.DOCX]

**Supplementary 1:** Search strategy for the PubMed database

| **Search** | **Query** |
| --- | --- |
| #21 | Search: **#3 AND #20** Sort by: **Most Recent** |
| #20 | Search: **#4 OR #7 OR #10 OR #13 OR #16 OR #19** Sort by: **Most Recent** |
| #19 | Search: **#17 OR #18** Sort by: **Most Recent** |
| #18 | Search: **anxiolytics misuse [Title/Abstract] OR cannabis misuse [Title/Abstract] OR hallucinogens misuse [Title/Abstract] OR inhalants misuse [Title/Abstract] OR opioids disorder [Title/Abstract] OR sedatives misuse [Title/Abstract] OR stimulants misuse [Title/Abstract]** Sort by: **Most Recent** |
| #17 | Search: **Drug Misuse [Title/Abstract]** Sort by: **Most Recent** |
| #16 | Search: **#14 OR #15** Sort by: **Most Recent** |
| #15 | Search: **gambling disorder [Title/Abstract] OR pathological gambling [Title/Abstract] OR problematic gambling [Title/Abstract] OR compulsive gambling [Title/Abstract] OR gambling addiction* [Title/Abstract] OR pathological gamblers [Title/Abstract] OR gamblers anonymous [Title/Abstract] OR gambling addicts [Title/Abstract]** Sort by: **Most Recent** |
| #14 | Search: **" Gambling "[Mesh]** Sort by: **Most Recent** |
| #13 | Search: **#11 OR #12** Sort by: **Most Recent** |
| #12 | Search: **suicide ideation [Title/Abstract]** Sort by: **Most Recent** |
| #11 | Search: **" suicide "[Mesh]** Sort by: **Most Recent** |
| #10 | Search: **#8 OR #9** Sort by: **Most Recent** |
| #9 | Search: **alcohol [Title/Abstract] OR drink* [Title/Abstract]** Sort by: **Most Recent** |
| #8 | Search: **" Ethanol "[Mesh]** Sort by: **Most Recent** |
| #7 | Search: **#5 OR #6** Sort by: **Most Recent** |
| #6 | Search: **tobacco [Title/Abstract] OR cigarette [Title/Abstract]** Sort by: **Most Recent** |
| #5 | Search: **" Smoking "[Mesh]** Sort by: **Most Recent** |
| #4 | Search: **" Health Risk Behaviors "[Mesh]** Sort by: **Most Recent** |
| #3 | Search: **#1 OR #2** Sort by: **Most Recent** |
| #2 | Search: **internet addiction [Title/Abstract] OR problematic internet use [Title/Abstract] OR internet addiction disorder [Title/Abstract] OR pathological internet use [Title/Abstract] OR excessive internet use [Title/Abstract] OR compulsive internet use [Title/Abstract] OR internet dependency [Title/Abstract] OR internet gaming addiction [Title/Abstract] OR internet gaming disorders [Title/Abstract] computer addiction [Title/Abstract] OR internet use disorder [Title/Abstract] OR smartphone addiction* [Title/Abstract]** Sort by: **Most Recent** |
| #1 | Search: **"Internet Addiction Disorder"[Mesh]** Sort by: **Most Recent** |
